# A performant bridge between fixed-size and variable-size seeding

**DOI:** 10.1186/s12859-020-03642-y

**Published:** 2020-07-23

**Authors:** Arne Kutzner, Pok-Son Kim, Markus Schmidt

**Affiliations:** 1 Department of Information Systems, College of Engineering, Hanyang University, 222 Wangsimni-ro, Seongdong-gu, Seoul, 04763 Republic of Korea; 2 Department of Information Security, Cryptology, and Mathematics, Kookmin University, 77, Jeongneung-ro, Seongbuk-gu, Seoul, 02707 Republic of Korea

**Keywords:** High-throughput sequence alignment, Minimizer, MEM, SMEM, FMD-index

## Abstract

**Background:**

Seeding is usually the initial step of high-throughput sequence aligners. Two popular seeding strategies are fixed-size seeding (*k*-mers, minimizers) and variable-size seeding (MEMs, SMEMs, maximal spanning seeds). The former strategy supports fast seed computation, while the latter one benefits from a high seed uniqueness. Algorithmic bridges between instances of both seeding strategies are of interest for combining their respective advantages.

**Results:**

We introduce an efficient strategy for computing MEMs out of fixed-size seeds (*k*-mers or minimizers). In contrast to previously proposed extend-purge strategies, our merge-extend strategy prevents the creation and filtering of duplicate MEMs. Further, we describe techniques for extracting SMEMs or maximal spanning seeds out of MEMs. A comprehensive benchmarking shows the applicability, strengths, shortcomings and computational requirements of all discussed seeding techniques. Additionally, we report the effects of seed occurrence filters in the context of these techniques.

Aside from our novel algorithmic approaches, we analyze hierarchies within fixed-size and variable-size seeding along with a mapping between instances of both seeding strategies.

**Conclusion:**

Benchmarking shows that our proposed merge-extend strategy for MEM computation outperforms previous extend-purge strategies in the context of PacBio reads. The observed superiority grows with increasing read size and read quality. Further, the presented filters for extracting SMEMs or maximal spanning seeds out of MEMs outperform FMD-index based extension techniques. All code used for benchmarking is available via GitHub at https://github.com/ITBE-Lab/seed-evaluation.

## Background

Most high-throughput read aligners [1–5] perform the following three steps: seeding [6, 7], seed processing (e.g. chaining) [8, 9] and dynamic programming [10, 11]. A sequence used as target for alignments is called reference. Reads aligned against such a reference are called queries. There are two techniques for seed computation: fixed-size seeding [12] and variable-size seeding [13, 14]. Fixed-size seeding is usually done via *k*-mers, which are perfect matches of size *k* between query and reference, or a space efficient variant of *k*-mers. Space efficiency with *k*-mers can be obtained by fixed sampling [15], where the index comprises every *m*-th *k*-mer merely, or minimizers [16]. A (*w*, *k*)-minimizer is a representative among a set of *w* consecutive *k*-mers that is determined using a scoring scheme based solely on the *k*-mers nucleotide sequences. The minimizer approach selects a subset of *k*-mers that still indicates all matches between reference and query of size ≥*w* + *k* − 1.

Variable-size seeding relies on maximal exact matches (MEMs) or subsets of them. A MEM [6] is a perfect match between query and reference that cannot be extended further in either direction. MEMs can be computed directly via some form of full-text search index as e.g. the FM-index [13, 14] that relies on a Burrows-Wheeler transform [17] based compressed representation of a suffix array. Two variants of the FM-index are the *n*-step FM-index [18] and the FMD-index [19]. The *n*-step FM-index extends *n* symbols at once for performance gains, while the FMD-index supports bi-directional search, via suffix-array interval decomposition, on sequences that comprise their own reverse complement. There are two subgroups within MEMs: SMEMs (super-maximal exact matches) [19] and maximal spanning seeds [2]. A SMEM is a MEM that is not enclosed by another MEM on the query. A MEM is a maximal spanning seed if and only if it comprises at least one query position that it is not covered by another longer MEM. This implies that a maximal spanning seed is always a SMEM but not the contrary.

**Fig. 1:**
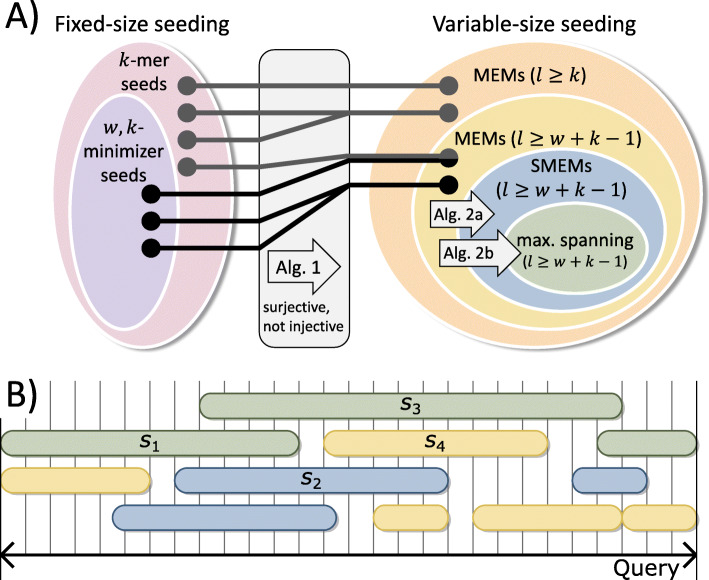
**a**) The hierarchies for fixed-size seeding (*k*-mer seeds and minimizer seeds) and variable-size seeding (MEMs, SMEMs, maximal spanning seeds) as well as the mapping among them. The ovals indicate subset relationships among the different seed sets. *l* denotes the length of an individual variable-size seed. Alg. 1 (Methods Section) constructs MEMs of size ≥*k* and ≥*w* + *k* − 1 out of *k*-mer seeds and (*w*, *k*)-minimizer seeds, respectively. Alg. 2a (Methods Section) in the methods section extracts SMEMs out of MEMs. Alg. 2b (Methods Section) computes maximal spanning seeds from MEMs (or SMEMs). **b**) visualizes the query positions and extensions of 11 instances of variable-size seeds. All displayed seeds are MEMs. Additionally, the blue and green seeds are SMEMs. Within the SMEMs, the green seeds are maximal spanning seeds. *s*_4_ is a MEM merely, because it is enclosed by *s*_3_. *s*_2_ is not enclosed by any other seed, but it is covered by longer seeds (*s*_1_ and *s*_3_). Therefore, *s*_2_ is a SMEM, but not a maximal spanning seed. *s*_1_ is not enclosed by any other seed and is the longest seed on the positions 0 to 7. Therefore, *s*_1_ is a maximal spanning seed

There exist proper subset relationships among variable-size seeding techniques as well as fixed-size seeding techniques. Further, there is a mapping between both groups as presented in Fig. 1. Alg. 1 (Methods Section) implements this mapping using a merge-extend strategy. The mapping is surjective, because for every MEM there is at least one corresponding *k*-mer seed. However, it is not injective, because several *k*-mer seeds can map to the same MEM. Trivially, the inverse mapping can be achieved by simply computing all *k*-size sub seeds of each MEM. (*w*, *k*)-minimizers are mapped to a subgroup within the set of MEMs.

An aligner can only find the correct location of a read if there are seeds for this location. Therefore, the seeding defines an upper bound for the maximal accuracy of an aligner. The mapping between *k*-mer seeds and MEMs (Fig. 1) shows an equivalence of fixed-size seeding and variable-size seeding with respect to such upper bound considerations.

We present and analyze an efficient algorithmic bridge for computing variable-size seeds out of fixed-size seeds. In contrast to previously proposed extend-purge strategies [15, 20–23], we follow a merge-extend strategy that avoids the creation of unnecessary MEM duplicates (Alg. 1, Methods Section). The detailed differences are discussed in the methods section and the superiority of our approach is shown in the results section. Further, we introduce two seed filters for performantly extracting SMEMs (Alg. 2a, Methods Section) and maximal spanning seeds from MEMs (Alg. 2b, Methods Section). Additionally, we introduce the notion correctness rate of a seed set as the number of nucleotides that a single seed contributes to an accurate alignment on average. In the results section, we show that SMEMs have a higher correctness rate than MEMs and that maximal spanning seeds have the highest correctness rate among all three. We investigate the impact of occurrence filtering techniques of state-of-the-art aligners with respect to the mapping and hierarchies shown in Fig. 1. Finally, we present use cases for our algorithmic approaches.

## Methods

**Fig. 2:**
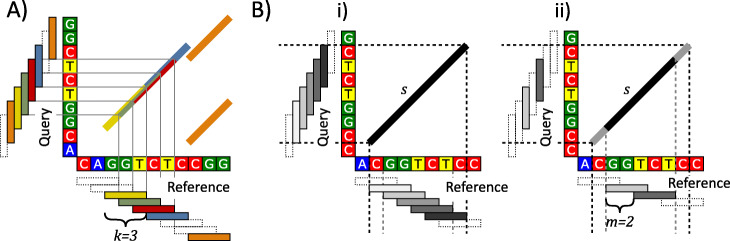
Several aspects of the MEM computation from *k*-mers. **a**) In a plane spanned by reference (x-axis) and query (y-axis), overlapping *k*-mers form MEMs. The yellow, green, red and blue 3-mers form a single long MEM that spans 6 nt. The two orange seeds are isolated and have no neighbors for merging. The dashed boxes on the reference represent 3-mer entries in the hash-table without a match on the query. The dashed boxes on the query visualize 3-mers that do not occur in the hash-Table. **b**) In subfigure i), the MEM *s* is formed by merging five *k*-mers (the grey boxes with solid outlines). Subfigure ii) shows the situation of i) for 2-step 3-mers, a subset of 3-mers that contains every second 3-mer merely. The first and last nucleotides of *s* are not covered by 2-step 3-mers and must be discovered by an additional extension

We first introduce our approach informally. Seeds can be visualized by drawing them as 45° lines in a plane that is spanned by reference (x-axis) and query (y-axis). The bottom-left coordinates of such a line are defined by the corresponding seed’s start positions on query and reference. The length of the line indicates the seed’s size. If *k*-mer seeds are visualized this way, some of them overlap or touch one another. Such overlapping or touching seeds can be merged into one single long seed. By performing all possible merges, we get the set of all MEMs for the inspected reference and query. Figure 2 shows an example of this seed merging. A detailed description of the plane based representation of seeds can be found in [2].

### Informal description

A major disadvantage of *k*-mers is the large size of the required hash-tables. For overcoming this problem, the concept of minimizers was introduced in [16]. Our proposed approach delivers MEMs for minimizers as well if the endpoints of all merged *k*-mers are additionally extended. However, the length of this extension has a strict upper bound. Therefore, the proposed technique delivers long, variable-size seeds out of *k*-mers. The practical value of our technique is an observed runtime superiority regarding seed computation over the FMD-index as well as a reduction of the number of seeds compared with minimizers.

We now present the construction of MEMs out of minimizers. For this purpose, we first describe our technique in the context of *k*-mers with step size of one. Next, we extend the concept to *m*-step *k*-mers, before we finally show that it is applicable for minimizers as well.

### Definitions and notations

Let Σ = {A, C, G, T} be the alphabet and let *W* ≔ *w*_0_*w*_1_…*w*_*n* − 1_ ∈ Σ^*n*^ be a non-empty word over Σ. A substring *w*_*x*_*w*_*x* + 1_…*w*_*y* − 1_ is denoted by *W*[*x*, *y*), with 0 ≤ *x* < *y* ≤ *n*; while a character access to *w*_*i*_ is denoted by *W*[*i*]. *W*^*k*^ ≔ {(*x*, *W*[*x*, *x* + *k*)) : 0 ≤ *x* ≤ |*W*| − *k*} denotes the set of all positioned *k*-mer sequences over *W*. Let *R*, *Q* ∈ Σ^+^ be a reference and query sequence, respectively. A seed is a perfectly matching section between *Q* and *R*, which is represented as a triple (*q*, *r*, *l*) with *q*, *r* ∈ *ℕ*_0_, where *q* and *r* are the starting positions on *Q* and *R*, respectively. *l* denotes the length of the seed (*l* ∈ *ℕ*_>0_). Let *s* = (*q*, *r*, *l*) be such a seed. The *δ*-value of *s* is defined as *r* − *q*. Using *R*〈*s*〉, we denote the subpart *R*[*r*, *r* + *l*) of the reference that belongs to *s*. Accordingly, *Q*〈*s*〉 denotes *Q*[*q*, *q* + *l*). Hence, a seed describes the equivalence of *R*〈*s*〉 and *Q*〈*s*〉.

For a reference *R* and query *Q*, the set of all MEMs is defined, in accordance with [6], as follows: For all pairs of reference-query positions (*x*, *y*), with *R*[*x*] = *Q*[*y*], 0 ≤ *x* < |*R*|, 0 ≤ *y* <∣*Q*∣, we get a MEM (*q*, *r*, *l*) as follows: Let *i* be the minimal value such that *R*[*x* − *i* − 1] ≠ *Q*[*y* − *i* − 1]. If there is no such value, then *i* = min(*x*, *y*). Using *i*, we set *q* = *y* − *i* and *r* = *x* − *i*. Further, let *j* be the minimal value such that *R*[*x* + *j*] ≠ *Q*[*y* + *j*]. If there is no such value, then *j* = min(|*R*| − *x*, |*Q*| − *y*). Using *j*, we set *l* = *j* + *i*. The above definition can repeatedly create identical MEMs. We assume that the set is purged of such duplicates.

Given a reference *R*, query *Q*, and their corresponding sets of *k*-mer sequences *R*^*k*^ and *Q*^*k*^: Let *C*^*k*^ ≔ {*Q*^*k*^ × *R*^*k*^} be the cross product of all positioned *k*-mer sequences on *Q* and *R*. Each pair ((*q*, *W*_*Q*_), (*r*, *W*_*R*_)) ∈ *C*^*k*^ with *W*_*Q*_ = *W*_*R*_ defines a *k*-mer seed (*q*, *r*, *k*). The set of all such seeds over *R*^*k*^ and *Q*^*k*^ is denoted by *Seeds*(*Q*^*k*^, *R*^*k*^). We call two seeds attached, if they have identical *δ*-values and overlapping or touching reference intervals. Trivially, this implies that attached seeds have overlapping or touching query intervals as well. Together, a set of attached *k*-mer seeds represents a larger region of equivalence between reference and query (see Fig. 2a). These larger regions correspond to the regions of equivalence described by the set of all MEMs.

### Algorithmic approaches – getting variable-size seeds from fixed-size seeds

Algorithmically, we can perform seed merging as follows: First, for a reference *R* and a query *Q*, we compute *Seeds*(*Q*^*k*^, *R*^*k*^) and store these seeds in an array *K*. Then, we sort *K* ascendingly according to the *δ*-values of all seeds. Seeds of equal delta value are sub-sorted according to their query position. By doing so, we guarantee that attached *k*-mers appear consecutively in *K*. In an iteration over *K*, we merge such attached *k*-mer seeds. The time complexity of these operations is bounded by the sorting of *K*.

If the proposed algorithmic approach is applied to a set of *k*-mer seeds, it delivers a set of MEMs. Figure 2a visualizes an example for the computation of three MEMs out of six 3-mer seeds. In the following section, we extend our approach towards minimizers.

### Extension of algorithmic approach to *m*-step *k*-mers

We first introduce *m*-step *k*-mers. For a word *W* and a given step-size *m*, the set of all positioned *k*-mer sequences (*x*, *W*[*x*, *x* + *k*)) fulfilling *x* mod *m* = 0 is called *m*-step *k*-mer sequences over *W* and is denoted by ${W}_m^k$. The *m*-step *k*-mers scheme is also referred to as fixed sampling [15, 23]. Alg. 1 implements our algorithmic approach in Pseudocode.

We store the *m*-step *k*-mer seeds $Seeds\left({Q}^k,{R}_m^k\right)$ in the array *K*. This altered seed set requires the following two additions to our algorithm: (1) Adjacent *m*-step *k*-mers on a *δ*-line can be separated by a gap if *m* > *k*. We try to close this gap via an extension process (lines 5 and 6). (2) The ends of a MEM are now not necessarily covered by k-mers anymore (e.g. Figure 2b ii). By iterating diagonally and comparing query and reference for equality, we maximally extend the seeds in *S* (lines 12–16).

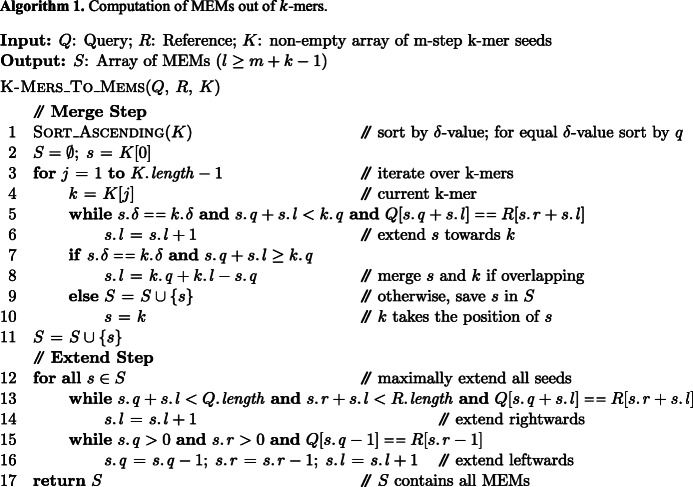



Please note, if we omit the merge step (lines 1–11) and maximally extend all *k*-mers (lines 12–16) immediately, we obtain all MEMs but with the following two significant disadvantages: (1) In the case of a single large match *M* that corresponds to a chain of attached *k*-mers, an immediate extension would extend all *k*-mers to the endpoints of *M*. Therefore, the size of *M* determines the amount of extensions required for each *k*-mer of the chain. (E.g. in Fig. 2a, the yellow, green, red and blue 3-mers would all be extended by 3 nt.) (2) A MEM containing multiple *k*-mers would be discovered multiple times. Therefore, we would need an additional purging scheme for getting rid of duplicates.

The above two points matter in the context of the variants of Alg. 1 proposed in [15, 20–22], which all extend *k*-mers before eliminating redundancy. We call their approach extend-purge strategy in contrast to the merge-extend strategy of Alg. 1.

### Formal proof

We now formally prove that our approach successfully retrieves all MEMs out of *m*-step *k*-mers.

**Theorem 1:***Let Q be a query, R be a reference and s* = (*q*, *r*, *l*) *be a MEM over Q and R. If l* ≥ *k* + *m* − 1*, then there is at least one k-mer seed covered by s in*$Seeds\left({Q}^k,{R}_m^k\right)$*.*

***Proof*:** Let *s'* = (*q′*, *r′*, *k*) be the sub-seed of *s* with *q'* = *q* + *x*, *r'* = *r* + *x* for the offset *x* = *m* − *r* mod *m* and the *k*-mer size *k*. By showing that *s*′ is in $Seeds\left({Q}^k,{R}_m^k\right)$, we prove the theorem. Let (*r'*, *R*〈*s′*〉) and (*q'*, *Q*〈*s′*〉) be two positioned *k*-mer sequences. Trivially, (*q'*, *Q*〈*s′*〉) ∈ *Q*^*k*^ and (*r'*, *R*〈*s′*〉) ∈ *R*^*k*^. We have to show that $\left({r}^{\prime },R\left\langle s^{\prime}\right\rangle \right)\in {R}_m^k$, which immediately follows from *r'* mod *m* = 0. Finally, we have to show *Q*〈*s′*〉 = *R*〈*s′*〉: Due to *s*, the words *R*〈*s*〉 and *Q*〈*s*〉 on reference and query must be equal. Considering *x* ≥ 0 and *x* + *k* − 1 ≤ *l*, the sub-words *R*〈*s′*〉 = *R*[*r* + *x*, *r* + *x* + *k*) and *Q*〈*s′*〉 = *Q*[*q* + *x*, *q* + *x* + *k*) must be equal as well. Altogether, *s′* results from (*q'*, *Q*〈*s′*〉) and (*r'*, *R*〈*s′*〉).

**Theorem 2:***For a set of m-step k-mer seeds K, Alg. 1 extends each seed s* = (*q*, *r*, *k*) ∈ *K with r mod m* = 0 *no more than m* − 1 *nt in either direction.*

***Proof:*** In lines 5, 6, 13 and 14 of Alg. 1, *s* cannot be extended more than *m* − 1 nt rightwards without merging with a neighboring seed *s′* = (*q* + *m*, *r* + *m*, *k*), since *Q*〈*s′*〉 = *R*〈*s'*〉, (*r* + *m*) mod *m* = 0 and *s'*.*δ* = *s*.*δ*. Accordingly, in lines 15 and 16 of Alg. 1, *s* cannot be extended more than *m* −1 nt leftwards without merging with a neighboring seed *s''* = (*q* − *m*, *r* − *m*, *k*), since *Q*〈*s′′* = *R*〈*s′′*〉, (*r* − *m*) mod *m* = 0 and *s''*.*δ* = *s*.*δ*. Please note: If *s* and *s*′′ are merged together, *s* is never left extended, since the merge happens during the right extension of *s′′*. Theorem 2. proves that Alg. 1 never extends a *k*-mer seed more than *m* −1 nt in either direction. Further, Alg. 1 merges all seeds that are connected during the extension process.

**Corollary 1.** For a reference *R* and a query *Q* and the *k*-mer seed set $Seeds\left({Q}^k,{R}_m^k\right)$, Alg 1. computes all MEMs with a size ≥*m* + *k* − 1 exactly once.

### Extension to minimizers and extraction of SMEMs and maximal spanning seeds

The proposed algorithmic approach for *m*-step *k*-mers can be applied to minimizers with a window of *m*, because the distance between two adjacent (*m*, *k*)-minimizers is always ≤*m* (as proven in [16]).

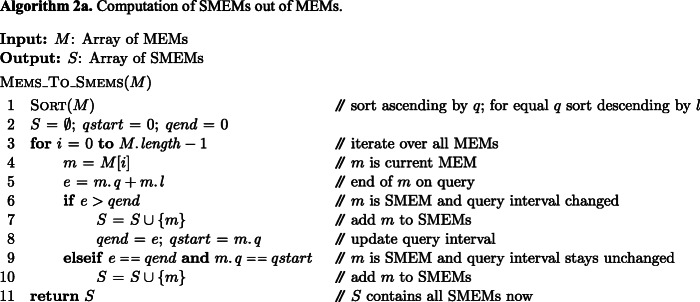



Algorithm 2a shows the Pseudocode for the extraction of SMEMs out of MEMs using a sorting followed by a single sweep over all MEMs. In order to prove the correctness of the algorithm, we characterize SMEMs (in accordance to [6, 19]) as follows: A SMEM is a MEM that is not enclosed by another MEM on the query. The above algorithm identifies all enclosed MEMs by iterating over them ordered by their start positions on the query.

The variable *qend* keeps the rightmost end-position of all MEMs visited during the iteration so far. For each iteration of the loop, we have: If the current seed *m* ends before *qend*, then *m* cannot be a SMEM, because the previously visited MEM that assigned *qend* encloses *m*. *qstart* memorizes the start position of the last SMEM. If start and end of the previous SMEM are equal to the current SMEMs start and end (line 9), then the current seed is a SMEM (line 10) that spans the same query interval as the previous one but has a different reference interval. Alg. 2a requires time *O*(*n* log *n*) for a set of *n* MEMs, assuming the initial sorting is done in time *O*(*n* log *n*).

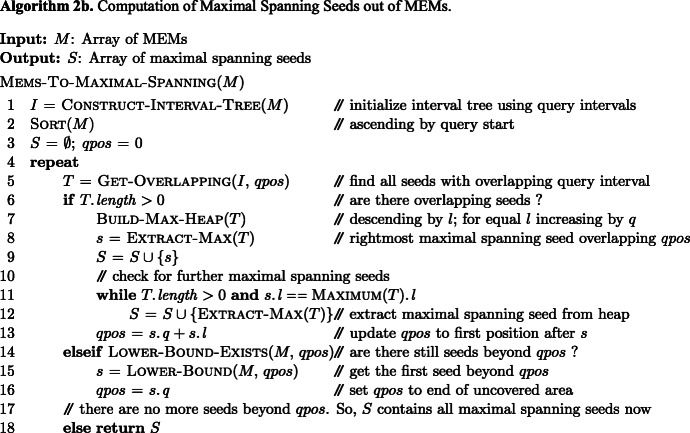



Within the MEMs, we identify the set of maximal spanning seeds as follows: A MEM is a maximal spanning seed if and only if it comprises at least one query position, where it is not covered by another longer MEM. Please note that the maximal spanning seeds always represent a subset of the SMEMs. (A detailed comparison of SMEMs and maximal spanning seeds can be found in [2]. Examples are given in Fig. 1b.)

We now informally describe the approach of Alg. 2b. A detailed analysis is given in Supplementary Note 1. In the central loop (lines 4–18), we visit all maximal spanning seeds exactly once via *qpos*. In each iteration, we distinguish between two situations:

1. *qpos* is within a set of seeds (Boolean condition in line 6 is true). We identify all maximal spanning seeds (line 8–12) by building a heap according to the seeds’ lengths (line 7) for all seeds overlapping *qpos* (line 5). If there are several maximal spanning seeds at *qpos*, then they are all identified in the while-loop (lines 11 and 12). Line 13 updates *qpos* for the next iteration. This guarantees that no maximal spanning seed is discovered twice or lost.
2. *qpos* is at the beginning of an area without seeds at all (Boolean condition in line 6 is false). In this case we move *qpos* to the first seed after this area (lines 14–16) or we return the computed set *S*, because there are no unprocessed seeds anymore (line 18). If *qpos* is moved to the start of a seed, we continue with case 1 in the next iteration.

**Theorem 4:***For a set of n MEMs, Alg. 2b) requires time O*(*n* log *n*), *assuming the sorting (line 2) and interval tree construction (line 1) happen in time O*(*n* log *n*).

***Proof*:** The central loop (lines 4–18) is executed ≤2*n* times. Further, lines 5, 14 and 15 require time *O*(log *n*) per iteration. In line 5, the accumulative size of all *T* is ≤*n*, because each MEM can only be part of a single iteration’s *T*. Therefore, all operations performed on *T* (lines 7, 8, 11 and 12) require an accumulative time of *O*(*n* log *n*).

## Results

We now prove the practical value of the proposed merge-extent technique by comparing its runtime behavior with the extend-purge strategy as well as FMD-index based seeding. Further, we introduce and discuss the correctness rate of various seed sets. Finally, we report about the effects of occurrence filtering in the context of seeding and present some practical use cases of our approach.

Our benchmarking relies on the human reference genome GRCh38.p12 for which we simulate PacBio circular consensus sequence (CCS) reads and continuous long sequence (CLR) reads. For read simulation, we use Survivor [24], where the error profiles and length profiles are sampled from the HG002 GIAB dataset [25]. We modulate the sampled error profile by applying a factor, called error rate, to the error probabilities forming the profile. In the diagrams, we start with perfect reads and increase the error rate until we meet the error profile of a specific kind of sequencer reads. The details of the modulation scheme together with a detailed description of the benchmarking environment are given in Supplementary Note 2. Because the superiority of the merge-extend strategy increases with increasing read length, our analysis focuses on long reads. However, Supplementary Note 4 contains an analysis for Illumina reads as well, where we use DWGSIM for read generation [26]. For comparing our merge-extend strategy with the extend-purge strategy, we rely on an implementation of the extend-purge strategy that is in accordance with the approaches presented in [15, 20–22]. Supplementary Note 3 describes this implementation in detail.

### Time evaluation

**Fig. 3:**
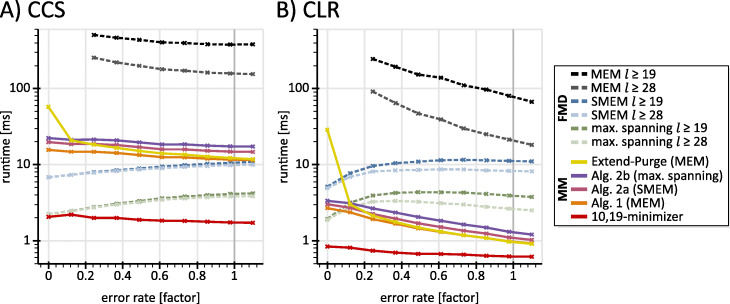
Time evaluation for minimizers, MEMs, SMEMs and maximal spanning seeds computed via the FMD-index and Alg.1, Alg. 2a and Alg. 2b. **a)** shows the runtimes for circular consensus sequencing (CCS) PacBio reads: The x-axis denotes a factor applied to the error profile of CCS PacBio reads, where zero corresponds to error free reads and one corresponds to reads following the measured error profile. Each point displays the average runtime for 1000 reads. The detailed scheme used for error rate computation is described in Supplementary Note 2. The pink and purple graphs include the times required for previous steps, i.e. the times for Alg. 1 and minimizer computation. Accordingly, the orange graph includes the time required for minimizer computation. The values for FMD-index based MEM computation below an error rate of 0.2 are omitted due to excessive runtimes. **b)** shows runtimes for continuous long PacBio reads (CLR)

We now analyze the runtime behavior of seeding techniques as visualized in Fig. 3. For minimizers, we purge all seeds that have more than 2000 reference position entries in the hash-table. For the FMD-index, we discard all suffix-array bi-intervals of size >2,000. The third subsection analyzes the effects of such filtering in detail. For computing MEMs via the FMD-index, we implement an algorithm based on [19, 27]. The Pseudocode of this algorithm together with a runtime comparison to the LCP-array based implementation of [27] is presented in Supplementary Note 7.

Our merge-extend strategy displays a general superiority over the extend-purge approach, where the superiority grows with increasing read quality. This can be explained as follows: For reads of high quality, the extend-purge strategy tends to extend multiple minimizers into the same MEM before purging all duplicates. In contrast, our merge-extend strategy computes each MEM only once. This problem worsens with increasing read length, because the extensions increase in size. Due to the expected improvements regarding the quality of long reads, these differences are of increasing relevance in practice. Further, the benchmarking shows that the FMD-index is not well suited for the computation of MEMs. Here, the runtimes grow almost exponentially with increasing read quality.

We now compare Alg. 2a and Alg. 2b (purple and pink curves of Fig. 3) with the FMD-index (blue and green curves of Fig. 3) in the context of the computation of SMEMs and maximal spanning seeds. With decreasing error rate, SMEMs and maximal spanning seeds increase in size but their numbers decrease. This behavior can be explained by the definitions of these seed sets via MEM enclosure on the query. On the contrary, k-mers and minimizers increase in number for decreasing error rates because of the increasing number of matching sections between query and reference. The runtimes of Alg. 2a and Alg. 2b reflect the number of minimizers (due to the application of Alg. 1 as pre-step), while the runtimes of the FMD-index reflect the size of the computed seed set (accumulated size of the suffix-array bi-intervals). This explains the reciprocal behavior of the curves from the respective approaches (purple and pink curves versus green and blue curves). Thus, for SMEM and maximal spanning seed computation, the preferred algorithmic approach depends on the quality of the reads. For continuous long PacBio reads (CLR), our measurements indicate that Alg. 2a and Alg. 2b outperform the FMD-index.

Although the extraction of maximal spanning seeds (purple curve) requires an interval tree, a heap and an initial sorting operation, it is only slightly slower than the SMEM filtering (pink curve). This can be explained by different behaviors of Alg. 2a and 2b in their central loops, where Alg. 2b merely iterates over maximal spanning seeds (and gaps among them), while Alg. 2a iterates over all MEMs.

### Correctness rates for various seed sets

**Fig. 4:**
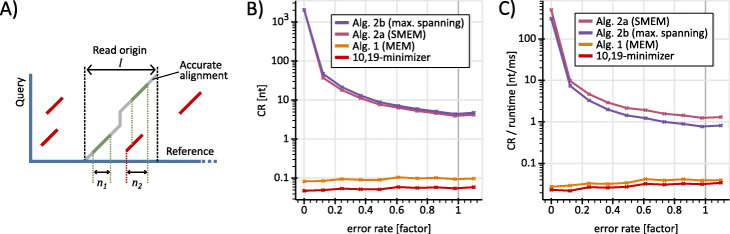
Correctness rate (CR) analysis. **a)** The figure visualizes Def. 1. The green seeds are contributing seeds (belong to the accurate alignment) and the red seeds are not contributing. For this example *n* = *n*_1_ + *n*_2_ and |*S*| = 6. **b)** analyzes the CR of CCS PacBio reads for (10, 19)-minimizer, MEMs, SMEMs and maximal spanning seeds. Each point in the diagram shows an average value for 1000 reads with an occurrence filter set to 2000. **c)** visualizes the rate of CR over the runtime, where the benchmarking environment is equal to b)

Let *r* be a read that originates from a reference interval *I*. If we compute seeds for *r*, we usually receive significantly more MEMs than SMEMs (due to the hierarchy shown in Fig. 1). Further, only a portion of the MEMs as well as SMEMs is expected to be correctly placed (lies within *I*) on the reference due to ambiguities. These correctly placed seeds cover subsections of *I*. The rate of this accumulative coverage of *I* over the size of the respective seed set delivers the average number of correctly placed nucleotides per seed. The higher this number of correctly placed nucleotides, the higher the average amount of a single seed’s information with respect to the accurate alignment. This amount of information is much higher for SMEMs than for MEMs (see Fig. 4), which explains the performance gains of aligners if they use SMEMs instead of MEMs. A seed set can cover a reference region multiple times by comprising overlapping seeds for this region. By using a coverage-based approach, we avoid a bias in our measurement towards such repeated information. We will now formalize our measurement of correct information and analyze it for other seed sets as well.

**Definition 1:***Let Q be a read that originates from a reference interval I. Let S be a set of seeds for Q. The correctness rate (CR) of S for Q is the rate n*/∣*S*∣*, where n is the number of nucleotides in I that are covered by seeds of S and* |*S*| *is the size of the set S.*

The CR expresses the number of nucleotides that a single seed contributes to an accurate alignment on average. Within the interval *I*, the CR does not distinguish between seeds that contribute to an accurate alignment and seeds that do not (e.g. the red seed in interval *I* in Fig. 4a is not contributing). These non-contributing seeds can erroneously increase the coverage *n* (e.g. the red seed in interval *I* erroneously increases the coverage). A more accurate definition of the correctness rate could be established on the foundation of contributing seeds merely. However, Survivor [24], the read generator used here, does not deliver sufficient information for identifying contributing seeds in *I* and so we have to rely on the proposed definition. Figure 4 visualizes our CR measurements for several seed sets using the various algorithmic approaches proposed here. Supplementary Note 8 justifies the chosen definition of the CR.

The curve for minimizers is always below the curve for MEMs (computed by Alg. 1). This can be explained as follows: Combining seeds (Alg. 1, lines 1–11) reduces the number of seeds while maintaining the coverage. Additionally, extending on both ends (Alg. 1, lines 12–16) increases the coverage while keeping the number of seeds. Hence, Alg. 1 can only improve the CR of a seed set. Fig. 4b) shows that the CR of SMEMs is always significantly higher than for MEMs. This confirms that SMEMs are a cleverly chosen subset of MEMs that is well suitable for alignments. Maximal spanning seeds always have the highest CR among all four seed sets. The rate of CR over runtime (Fig. 4c) indicates that the tradeoff between additional runtime and improved CR is in favor of Alg. 2a and Alg. 2b over MEMs and minimizers.

Our measurement express the CR of a seed set and not its capability to allow accurate alignments. The proposed algorithmic approaches can only deliver seeds that are discoverable via minimizers and nothing beyond.

### Filter and effects

**Fig. 5:**
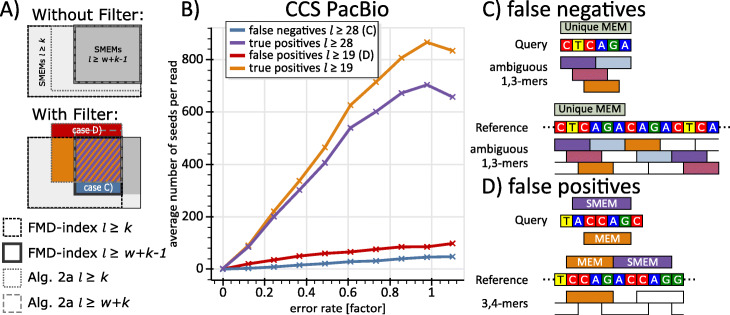
Analysis and effects of occurrence filtering. **a)** The four kinds of boxes visualize subset relations among the SMEMs computed by the FMD-index and Alg. 2a, where Alg. 2a uses (*w*, *k*)-minimzers. The light and dark shaded areas represent the sets of all SMEMs with *l* ≥ *k* and *l* ≥ *w* + *k* − 1, respectively. The top diagram shows the subset relations without occurrence filtering, while the bottom diagram displays the situation with occurrence filtering. For *l* ≥ *w* + *k* − 1 and without filters, Alg. 2a and the FMD-index deliver equal sets of SMEMs (the two innermost boxes in the top diagram). For *l* ≥ *k*, Alg. 2a misses some SMEMs discovered by the FMD-index (the dotted box within SMEMs *l* ≥ *k*). In the presence of occurrence filtering, all boxes shrink in size. Further, Alg. 2a computes false negatives (the blue box, case c) and false positives (the red box, case d). **b)** The diagram delivers a positive-negative classification of Alg. 2a using the seeds remaining after occurrence filtering with the FMD-Index as ground truth for (10, 19)-minimizers on the human genome. The four curves correspond to the sizes of the four respectively colored areas in a) for various error rates. The x-axis represents the error rate of reads as described in Fig. 3. Each dot shows the average value for 1000 CCS PacBio reads generated using Survivor [24]. **c)** There is a MEM covering the unique sequence CTCAGA on query and reference. Assuming, the occurrence threshold for minimizers is set to two, the four colored 1,3-mers are purged. In this case, the MEM cannot be discovered by Alg. 2a. Using the FMD-index, this MEM is discovered directly and not purged by the occurrence filter, since it occurs only once. **d)** explains the generation of SMEMs that are false positives. The purple SMEM cannot be discovered using the 3,4-mers, since no 3,4-mer is contained within the seed. However, the orange MEM can be discovered using the orange 3,4-mer. Now, Alg. 2a will not delete this MEM, since it is not enclosed by any other seed

Most aligners (e.g. Minimap 2 [3], MA [2], BWA-MEM [4]) use a filtering scheme in order to cope with repetitive regions of the genome. Using a threshold, seeds of query intervals that show an excessive amount of occurrences (i.e. a high ambiguity) on the reference are purged during seeding. However, minimizers and FMD-index apply these filters on different stages of the seeding process. The FMD-Index filters after the completion of the extension (using the size of the suffix-array intervals), while minimizers filter using the size of the hash-table buckets. In Fig. 5, we evaluate the effect of this difference in the context of the human genome.

For the evaluation of the effects of occurrence filtering as shown in Fig. 5, we use Minimap 2 for the minimizer computation of Alg. 2a and MA for the FMD-index based SMEM computation. Here, the occurrence filters of both aligners are set as follows: For Minimap 2 we use *‘-f 0 --min-occ-floor 2000’* for forcing the inclusion of all *k*-mers occurring 2000 times or less on the reference and for switching off the fraction driven *k*-mer dropping (*−f* parameter controlled). MA’s *‘Maximal Ambiguity’* parameter is set to 2000. Therefore, we drop seeds occurring more than 2000 times on the reference with both aligners.

Without filtering, the theoretical equivalence implies that the red and blue curves of Fig. 5b must constantly be zero. The blue line, SMEMs found via the FMD-index merely, results from situations like the one depicted in Fig. 5c: There exist 19-mers that exceed the occurrence threshold while the respective maximally extended seed does not. However, as shown in Supplementary Note 5, such seeds have a low correctness rate and so they are not expected to contribute to an accurate alignment.

Further, there are SMEMs that are found via Alg. 2a but not via the FMD-index. These SMEMs are false positives and their appearance is explained in Fig. 5d: Due to the absence of minimizers, a SMEM stays undiscovered. Instead, the largest MEM inside the query interval of that SMEM appears as a false positive SMEM now. These false positives have a very low correctness rate as shown in Supplementary Note 5. Please note, without filtering and for *l* ≥ *w* + *k* − 1 (for (*w*, *k*)-minimzers) such false positives vanish since the absence of minimizers becomes impossible.

Supplementary Note 5 contains a corresponding occurrence filter analysis for maximal spanning seeds as well as CLR PacBio reads.

## Discussion

Alg. 1 represents a surjective and not injective mapping from *k*-mers to MEMs (see Fig. 1). Therefore, informally spoken, everything that can be discovered with *k*-mers (fixed-size seeding) can also be discovered using MEMs (variable-size seeding) and vice versa. This implies that there is no theoretical superiority of one of these seeding techniques in contrast to assumptions made by us [2] and others [3]. A sophisticated chaining of minimizers should deliver the same alignment accuracy as a sophisticated chaining of MEMs.

Let |*S*|_⊤_ and |*S*|_⊥_ be the absolute number of correct and incorrect seeds in a set of seeds *S*, respectively. Then, we get the following hierarchies:

$$ {\left|k-\mathrm{mers}\right|}_{\top}\ge {\left|\left(w,k\right)-\mathrm{minimizers}\right|}_{\top}\ge {\left|\mathrm{MEMs}\right|}_{\top}\ge {\left|\mathrm{SMEMs}\right|}_{\top}\ge {\left|\max .\mathrm{spanning}\ \mathrm{seeds}\right|}_{\top },{\left|k-\mathrm{mers}\right|}_{\perp}\ge {\left|\Big(w,k\Big)-\mathrm{minimizers}\right|}_{\perp}\ge {\left|\mathrm{MEMs}\right|}_{\perp}\ge {\left|\mathrm{SMEMs}\right|}_{\perp}\ge {\left|\max .\mathrm{spanning}\ \mathrm{seeds}\right|}_{\perp }, $$

where MEMs, SMEMs and maximal spanning seeds are of size ≥*w* + *k* − 1. However, our benchmarking suggests a reverse hierarchy regarding the correctness rate (see Results Section). Hence, with maximal spanning seeds, an aligner will use the seed set with the highest correctness rate, but it is at high risk of missing an accurate alignment due to the lack of correct seeds. In contrast, with *k*-mers an aligner has access to the largest number of correct seeds but struggles to cope with the excessive amount of incorrect seeds.

Naturally, each approach that processes a set of seeds (e.g. chaining, clustering) has a time complexity driven by the number of seeds to be processed (e.g. non-heuristic chaining can have a squared worst case complexity [3]). Therefore, the above hierarchies for the number of correct (and incorrect) seeds within sets of seeds also imply hierarchies for the times required to process these sets (processing *k*-mers is the most expensive, while processing maximally spanning seeds is the cheapest). If the runtime of one of our algorithms is less than the time required to process the respectively larger seed set (e.g. from minimizers to MEMs with Alg. 1), we get an overall runtime advantage. Here the accuracy can suffer with SMEMs (Alg. 2a) and maximal spanning seeds (Alg. 2b), since the amount of correct seeds is reduced, as mentioned above.

If a seed processing algorithm can deliver all seeds sorted by their *δ*-values, the sorting operation of Alg. 1 (line 1, sorting by *δ*-value) is not required anymore. This gives Alg. 1 a linear runtime complexity and so all MEMs can be computed in linear time. An example of a seed processing strategy that can exploit this aspect of Alg. 1 is the Strip of Consideration proposed in [2].

The hash tables required for minimizer seeding can be computed in short time by a single scan over the reference. Therefore, minimizer indices are well suited for the “on-the-fly” computation of single-use indices for subsections of the human genome. Given such a single-use index, Alg. 1, Alg. 2a and Alg. 2b can quickly compute MEMs, SMEMs and maximally spanning seeds for these subsections. The computed seeds, in turn, can improve an alignment’s accuracy or bridge gaps that would otherwise require Dynamic Programming.

FMD-Index computation can be boosted by exploiting concurrency. Trivially, such introduction of concurrency is possible in the context of minimizers as well. We limit our benchmarking to a single thread in order to get a fair comparison of both techniques.

Our analysis bases on the human genome and a limited range of parameter combinations. A more comprehensive study could yield more insights here. Further, the design of more sophisticated heuristics for occurrence filtering of minimizers deserves additional research.

## Conclusion

Our novel algorithmic approaches allow the generation of SMEMs and maximal spanning seeds using minimizers. Particularly for long reads of high quality, our merge-extend strategy for MEM computation is faster than existing extend-purge approaches. In the context of aligner design, the proposed hierarchies within fixed-size seeding and variable-size seeding together with their respective correctness rates can be used for choosing an appropriate seeding technique (e.g. choosing SMEMs over MEMs is expected to decrease runtime but for the price of a slightly worse accuracy). The reported impact of occurrence filters helps assessing their effects with respect to the accuracy runtime tradeoff of alignments. Summarily, our presented algorithms and insights are valuable in the context of designing and using aligners.

## Supplementary information

**Additional file 1: Supplementary Note 1.** Detailed analysis of Alg. 2b. **Supplementary Note 2.** Detailed description of the read simulation and error rate. **Supplementary Note 3.** Detailed description of the extend-purge scheme. **Supplementary Note 4.** Results for Illumina reads. **Supplementary Note 5.** Comprehensive Occurrence Filter Effects. **Supplementary Note 6.** Runtime Evaluation. **Supplementary Note 7.** MEM computation using the FMD-index. **Supplementary Note 8.** Justification of Correctness Rate’s Definition

## Data Availability

All code and datasets supporting the conclusions of this article are available via the GitHub repository, https://github.com/ITBE-Lab/seed-evaluation under the MIT License. The evaluation tools are realized using Python 3.6, where time critical components (minimizer computation, proposed algorithms etc.) are implemented in C++ 17. All code runs under Debian Linux 9.12 (stretch).

## Abbreviations

CCS: Circular consensus sequencing
CLR: Continuous long read
CR: Correctness rate
DP: Dynamic programming
MEM: Maximal exact match
SMEM: Supermaximal exact match
