# Supplementary material for: A performant bridge between fixed-size and variable-size seeding
Source: BMC Bioinformatics. 2020 Jul 23;21:328. doi: 10.1186/s12859-020-03642-y (PMC7376731; doi:10.1186/s12859-020-03642-y)
Supplement: Supplementary file 1 — Additional file 1: Supplementary Note 1. Detailed analysis of Alg. 2b. Supplementary Note 2. Detailed description of the read simulation and error rate. Supplementary Note 3. Detailed description of the extend-purge scheme. Supplementary Note 4. Results for Illumina reads. Supplementary Note 5. Comprehensive Occurrence Filter Effects. Supplementary Note 6. Runtime Evaluation. Supplementary Note 7. MEM computation using the FMD-index. Supplementary Note 8. Justification of Correctness Rate’s Definition [file 12859_2020_3642_MOESM1_ESM.docx]

A performant bridge between fixed-size and variable-size seeding

Arne Kutzner^1^ Pok-Son Kim^2^ and Markus Schmidt^1,*^

^1^ Department of Information Systems, College of Computer Science, Hanyang University, 222 Wangsimni-ro, Seongdong-gu, Seoul, 04763, Republic of Korea
^2^ Department of Mathematics, College of Science and Technology, Kookmin University, 77, Jeongneung-ro, Seongbuk-gu, Seoul, 02707, Republic of Korea

* To whom correspondence should be addressed. Email: [schmidtm@hanyang.ac.kr](mailto:schmidtm@hanyang.ac.kr)

**Supplementary Information**

# Supplementary Note 1 - Detailed analysis of Alg. 2b.

**Lemma 1:** Let $M$ and $S$ be the set of all MEMs and all maximal spanning seeds over a given reference and query, respectively. Alg. 2b computes$S$ out of $M$.

**Proof:**

We show the correctness of the following loop invariant for all iterations of the central loop (lines 4 -18):

**Loop invariant:** At position $qpos$, we collect all maximal spanning seeds that encompass $qpos$. $S$ contains all max. spanning seeds that end before $qpos$. There is no seed that occurs twice in $S$.

**Initialization:** For $qpos=0$, we have $S=\emptyset$. ($S$ is empty, because it comprises all seeds left of $qpos$ and there cannot be a seed ending before position 0.)

**Maintenance:** We have to distinguish 2 cases at $qpos$:

1. No seed overlaps $qpos$: In this case, there is no seed to be collected; $S$ stays unchanged. Lines 14-16 move $qpos$ to the start of the next seed. (The next following case must be case 2.)
2. There are seeds overlapping $qpos$: Let $O$ be the set of those seeds. $O$ is computed in line 5. By definition, a seed is maximal spanning if and only if it comprises at least one query position, where it is not covered by another longer MEM. At position $qpos$, this condition holds for all seeds of $O$ that are added to $S$ in lines 7-12 via the priority queue (max heap). Hence, we collect all max. spanning seeds overlapping $qpos$.
   We now have to show that the next stop of $qpos$ is chosen so that no max. spanning seed is selected more than once (a) or skipped (b):
   Let $s:=\left( q,r,l \right)$ be the seed extracted in line 8.
   1. Due to the heap ordering, $s$ is the rightmost extending seed among all max. spanning seeds in $O$. By setting $qpos$ to the first position after $s$ (line 13), we will never collect any seed twice.
   2. By contradiction, we prove that no max. spanning seed is skipped: Assume these is a max. spanning seed $s':=\left( q^{'},r^{'},l^{'} \right)$ that is skipped, i.e. $s^{'}$overlaps neither $qpos$ nor $q+l$ (the position of $qpos$ in the next iteration). Hence, $qpos<q'$ and $q^{'}+l^{'}\leq q+l$. Since we extracted $s$ at the position $qpos$, we have $q\leq qpos$. Therefore $s'$ is fully enclosed by $s$ ($q<q^{'}<q^{'}+l^{'}\leq q+l$) and cannot be a max. spanning seed.

**Termination:** $qpos$ is increased in every iteration by at least one nt. As soon as $qpos$ is past the end of the query, there cannot be any overlapping seed or any seed to the right of $qpos$. Then, line 18 terminates the central loop.

# Supplementary Note 2 - Detailed description of the read simulation and error rate

All benchmarking is done using the human reference genome GRCh38.p12 (GenBank assembly accession: GCA_000001405.27). For the simulation of PacBio circular consensus sequence (CCS) reads and continuous long sequence (CLR) reads, we use the program Survivor [1], version v1.0.5-14-g18bf070. For the simulation of 250nt Illumina reads, we rely on the program DWGSIM [2], version 0.1.12-2-g39a1bbb. All diagrams denoting an “error rate” on the x-axis (Fig. 3, 4 and 5 of the main manuscript) are computed as follows:

At the x-axis position labeled ‘1’, we show measurements for the “standard error rate” of the benchmarked type of reads (PacBio CCS etc.). The standard error rate is chosen as follows:

- For Survivor CLR PacBio reads, we use the error profile provided in the GitHub repository of Survivor.
- For Survivor CCS PacBio reads, the profile is measured using Survivor and the CCS 10kb PacBio reads of the HG002 individual in the GIAB dataset.

(<ftp://ftp-trace.ncbi.nlm.nih.gov/giab/ftp/data/AshkenazimTrio/HG002_NA24385_son/PacBio_CCS_10kb/>)

- All Illumina reads are created using the standard setting of DWGSIM.

At the x-axis position labeled ‘0’, error free reads are used for benchmarking. The fractional values on the x-axis denote factors that are applied to the standard error rate. For values smaller than one, the error rate is decreased; otherwise, the error rate is increased. In more detail, this is done as follows:

- DWGSIM: The modulation of the error rate happens by multiplying the x-axis factor with 0.001 (default value of ‘-r’ rate of mutations parameter) and using the result as the new rate of mutations.
- Survivor: Using the error profile for the standard error rate, we create a specific error profile for a given factor $f$ as follows: In Survivor, error profiles are represented using tables consisting of 6 columns:
  1. a position value $p$
  2. probability $P_{stop}\left( p \right)$ that a read ends at $p$
  3. probability $P_{match}\left( p \right)$ for a match at $p$
  4. probability $P_{mismatch}\left( p \right)$ for a mismatch at $p$
  5. probability $P_{ins}\left( p \right)$ for an insertion at $p$
  6. probability $P_{del}\left( p \right)$ for a deletion at $p$

For each row, we compute factor specific values $P^{'}\left( p \right)$ as follows:

${P'}_{stop}\left( p \right)=P_{stop}\left( p \right)$

${P'}_{match}\left( p \right)=1-(1-P_{match}\left( p \right)*f)$

${P'}_{mismatch}\left( p \right)=P_{mismatch}\left( p \right)*f$

${P'}_{ins}\left( p \right)=P_{ins}\left( p \right)*f$

${P'}_{del}\left( p \right)=P_{del}\left( p \right)*f$

All benchmarking with respect to the FMD-index is done using the FMD-index implementation of MA (version 1.1.1-a7a0989) [3]. Computing MEMs using the FMD-index is done via an adoption of the algorithm in [4] from the FM-index to the FMD-index. The generation of minimizers is measured using code of Minimap 2 (version 2.12-r829) [5].

In the context of our benchmarking for seeding, we measure the time required for the actual seed production. For minimizers, this includes the time required for the minimizer computation as well as the time required for the hash table lookup of all reference positions. For the FMD-index, this includes the time required for the extension as well as the time required to extract the reference positions of seeds form the suffix array.

All benchmarking is done on an *AMD Ryzen Threadripper 1950X 16-Core Processor* with 128 GB RAM. For compilation, we rely on gcc (version 6.3.0). As software environment, we use Debian GNU/Linux with a 4.9.0 kernel.

All code is available as open source at: https://github.com/ITBE-Lab/seed-evaluation

# Supplementary Note 3 – Detailed description of the extend-purge scheme

The above algorithm implements the extend-purge scheme for MEM computation. It is used for benchmarking the extend-purge approaches and is in accordance with previously published variants of this scheme for MEM computation [6-9]. The cited works mainly focus on strategies for cleverly selecting seeds for the set $K$. These selection strategies could be applied in the context of Alg. 1 (merge-extend based MEM construction) of the main manuscript as well. Hence, we do not incorporate or analyze these strategies here.

The algorithm consists of two segments:

Segment 1 (lines 1-5):

The for-loop visits all seeds in $K$ and maximally extends them to the left and right via the corresponding while-loops. This part of the algorithm is equal to the extension step in Alg. 1 (lines 12-16) of the main manuscript.

Segment 2 (line 6 -12):

This part deletes all duplicates resulting from the extension. First, we sort all seeds so that identical seeds become neighbors. Then we iterate over the sorted seeds and purge all seeds that are identical to their predecessor.

A comparison of the merge-extend and extend-purge strategies shows:

- The merge-extend benefits from long and “clean” reads, because the purge-extend strategy has to perform longer extensions for more minimizers ($k$-mers) here.
- The extend-purge strategy gains advantage over the merge-extend strategy in cases of few extensions because the purge step is computationally slightly less expensive than the merge step.

The above two observations are practically supported by the runtimes of Fig. 3 of the main manuscript as well as Supplementary Note 4.

# Supplementary Note 4 – Results for Illumina reads

## a) Time Evaluation

The diagram shows a runtime evaluation for generate Illumina reads (see Supplementary Note 2) similar to the runtime evaluation for PacBio CCS and CLR reads in Fig. 3 of the manuscript. The left diagram displays correctness rates for an occurrence filter setting of 2000 and the right diagrams for a setting of 200. The curves show a quite constant behavior, since the error rate of Illumina reads is quite low. As expected, the merge-extend (orange curve) and extend-purge (yellow curve) runtimes are quite close to each other due to the short size of Illumina reads. Compared to Fig. 3, the computation of maximal spanning seeds using the FMD-index is faster than all other FMD-index based seeding approaches. This is in accordance with the observation for maximal spanning seeds on error free reads in Fig. 3B).

## b) Correctness Rate Analysis

The diagram shows the correctness rate analysis for Illumina reads similar to the analysis for PacBio CCS reads in Fig. 4B). The left diagram displays correctness rates for an occurrence filter setting of 2000 and the right diagrams for a setting of 200. In accordance with the theoretical considerations with respect to the correctness rate, there is no change among the order of the curves for the respective kinds of seed sets (minimizer, MEM, SMEM, maximal spanning seeds). As for the runtime analysis, the curves express a quite constant behavior because the standard error for Illumina reads is quite low.

# Supplementary Note 5 – Comprehensive Occurrence Filter Effects

SMEM

Maximal spanning seeds

Occurrence filter = 2,000

Occurrence filter = 200

The above diagrams extend the occurrence filter analysis shown in Fig. 5 of the main manuscript:

The diagrams of the top two rows are computed using an occurrence filter setting of 2,000, while the bottom two rows are for an occurrence filter setting of 200. The left two columns show an analysis for SMEMs, while the right two columns show an analysis for maximal spanning seeds. The columns A) and C) display diagrams for CCS PacBio reads, while the columns B) and D) are for PacBio CLR reads. The y-axes in the first and third row express “number of seeds” as in Fig. 5B) of the manuscript. In the diagrams of the second and fourth row, each point shows the average correctness rate (CR) of all seeds of its corresponding point (same color, same x-axis position) in the respective diagram above. For low error rates (< 0.1), the CR values are omitted for the following reasons:

- For true positives, the CR tends towards the average read length.
- For false positives and false negatives, the lack of seeds turns the CR meaningless.

The benchmarking environment and occurrence filter settings are described in the results section.

We first discuss the diagrams for CCS reads:

The curves for SMEMs and maximal spanning seeds are quite similar. Further, the number of false-positives (seeds erroneously identified as SMEMs by Alg. 2a or identified as maximal spanning seeds by Alg. 2b) and false-negatives (missed by Alg. 2a or Alg. 2b) are quite low. The CR diagrams indicate that the false-negatives and false-positives are not relevant in the context of accurate alignments due to their low CR.

We now discuss the CLR reads:

Because CLR reads have a worse quality than CCS reads, seeds are expected to be of shorter size than for CCS reads. With the decreasing size of seeds, the risk of a seed to occur multiple times on the reference increases. If the number of occurrences exceeds a given threshold, a seed is purged. This causes the decreasing behavior of the orange and purple curves in the CLR diagrams, starting at an error rate of 0.5. Additionally, the CR of the seeds close to an error rate of 1 is low for CLR reads compared with CCS reads. The number of false positives and false negatives for CLR reads does not differ significantly from the corresponding values for CCS reads with the exception of false positives in subfigure D) and H). These false-positives need to be purged by the seed processing (chaining etc.).

Despite different occurrence filter settings, the relations among the curves stay mostly unaltered. For example, the diagrams D) and H) are different with respect to the absolute $y$-values of all curves but their shapes and the relations among them (cross points, peak points) stay equal.

# Supplementary Note 6 – Runtime Evaluation

The below diagrams show the analysis of Fig. 3 for the occurrence filter set to 2,000 (subfigures A and B) and 200 (subfigures C and D). (Details of the occurrence filtering are described in section 3.3 of the manuscript.)

For the reduced occurrence filter setting, our algorithmic approaches show a general superiority over the FMD-index based seed computation. The reason for this shift towards Alg.1, Alg. 2a and Alg. 2b is the ambiguity of the genome. SMEMs and maximally spanning seeds are expected to be significantly less ambiguous than minimizers due to their higher correctness rate (see Section 3.2 of the main manuscript). Hence, the lower setting of the occurrence filter shows more effect on Alg. 1 than on FMD-index based seeding. Further, the superiority of the merge-extend strategy over the extend-purge strategy increases. This can be explained as follows: Ambiguous, wrongly placed seeds tend to be isolated. Such isolated seeds have no neighbors to be merged with; they are simply extended (and so both strategies perform the same amount of work). The lower occurrence filter reduces the number of such outliers and therefore the initial merging of our approach is applied more frequently. This in turn leads to the observed runtime-gains of our approach.

Additionally to the manuscript, all diagrams show the times required for the suffix array extension with the FMD-index. (Dashed lines labeled “Extension”.) For MEMs, the extraction of seeds is very expensive in relation to the extension of seeds, while this is not the case for SMEMs and maximal spanning seeds. This is explained by the large quantity of MEMs in comparison to SMEMs and maximal spanning seeds. Further, a decreasing error rate worsens the situation as explained in the main manuscript (section 3.1, third paragraph). However, in most cases, the extension alone is slower than the full extraction via Alg. 1, Alg. 2a and Alg. 2b.

# Supplementary Note 7 – MEM computation using the FMD-index

The definition of the $C$-array (Accumulative count array of the FMD-index) as well as the definition of BACKWARDEXT can be found in [10]. Generally, the above algorithm implements the ideas for MEM-computation of Ohlebusch et al. [4] and uses the bidirectional extension opportunities of the FMD-index instead of a LCP-array. The usage of the bidirectional extension, in turn, strongly resembles “Algorithm 5: Finding SMEMs” of Heng Li in [10].

Below we compare the runtimes of our FMD-index based implementation (FINDING-MEMs) with the LCP-array based implementation (BackwardMEM) of Ohlebusch et al.:

The above runtimes are computed using the benchmarking environment reported for CCS PacBio reads in Supplementary Note 2, where read generation and indices are limited to Chromosome 1 (The limitation to Chromosome 1 is required for preventing segmentation faults occurring in BackwardMEM if used with the whole genome). Since BackwardMEM does not support occurrence filtering, we disable the occurrence filtering for our approach as well. The runtimes indicate that the FMD-index seems to be better suited for a suffix array based MEM computation than LCP-arrays.

# Supplementary Note 8 – Justification of Correctness Rate’s Definition

The above figure corresponds to Fig. 4B) of the manuscript. Instead of correctness rates, it displays the ratio of correct seeds to all seeds on the y-axis. As with the definition of the correctness rate, a seed is considered “correct”, if it overlaps a read’s origin interval $I$ (see Fig. 4A).

The above figure indicates that, compared with MEMs, minimizers are the better seed set with respect accurate alignments due to their better ratio. However, this contradicts the theoretical considerations in section 3.2 of the manuscript. Minimizer gain the observed superiority in the above diagram, since each match of size $\geq w+k-1$ (for $(w,k)$-minimzers) between query and reference is covered by a chain of (overlapping) minimizer that represent a single MEM in their merged form (see Fig. 2 A for a $k$-mer based example of the problem). For avoiding the resulting bias in favor of minimizers, the correctness rate is defined using the coverage of the interval $I$ on the reference.

# Supplementary References

1. Jeffares, D.C., et al., *Transient structural variations have strong effects on quantitative traits and reproductive isolation in fission yeast.* Nature Communications, 2017. **8**(1): p. 14061.

2. Homer, N., *Dwgsim: whole genome simulator for next-generation sequencing.* GitHub repository, 2010.

3. Schmidt, M., K. Heese, and A. Kutzner, *Accurate high throughput alignment via line sweep-based seed processing.* Nature Communications, 2019. **10**(1): p. 1939.

4. Ohlebusch, E., S. Gog, and A. Kügel. *Computing Matching Statistics and Maximal Exact Matches on Compressed Full-Text Indexes*. 2010. Berlin, Heidelberg: Springer Berlin Heidelberg.

5. Li, H., *Minimap2: pairwise alignment for nucleotide sequences.* Bioinformatics, 2018. **1**: p. 7.

6. Darling, A.C., et al., *Mauve: multiple alignment of conserved genomic sequence with rearrangements.* Genome research, 2004. **14**(7): p. 1394-1403.

7. Liu, B., et al., *deBGA: read alignment with de Bruijn graph-based seed and extension.* Bioinformatics, 2016. **32**(21): p. 3224-3232.

8. Liu, Y., L.Y. Zhang, and J. Li, *Fast detection of maximal exact matches via fixed sampling of query K-mers and Bloom filtering of index K-mers.* Bioinformatics, 2019. **35**(22): p. 4560-4567.

9. Khiste, N. and L. Ilie, *E-MEM: efficient computation of maximal exact matches for very large genomes.* Bioinformatics, 2014. **31**(4): p. 509-514.

10. Li, H., *Exploring single-sample SNP and INDEL calling with whole-genome de novo assembly.* Bioinformatics, 2012. **28**(14): p. 1838-1844.
